# Empirical estimates of prostate cancer overdiagnosis by age and prostate-specific antigen

**DOI:** 10.1186/1741-7015-12-26

**Published:** 2014-02-11

**Authors:** Andrew J Vickers, Daniel D Sjoberg, David Ulmert, Emily Vertosick, Monique J Roobol, Ian Thompson, Eveline AM Heijnsdijk, Harry De Koning, Coral Atoria-Swartz, Peter T Scardino, Hans Lilja

**Affiliations:** 1 Department of Surgery (Urology), Memorial Sloan-Kettering Cancer Center, New York, NY, USA; 2 Department of Epidemiology and Biostatistics, Memorial Sloan-Kettering Cancer Center, New York, NY, USA; 3 Laboratory Medicine and Medicine (GU-Oncology), Memorial Sloan-Kettering Cancer Center, New York, NY, USA; 4 Department of Urology, Erasmus University Medical Center, Rotterdam, Netherlands; 5 Department of Public Health, Erasmus University Medical Center, Rotterdam, Netherlands; 6 Departments of Laboratory Medicine and Clinical Sciences in Malmö, Lund University, University Hospital UMAS, Malmö, Sweden; 7 The Cancer Therapy and Research Center, University of Texas Health Science Center at San Antonio, San Antonio, TX, USA; 8 Nuffield Department of Surgical Sciences, University of Oxford, Oxford, UK

**Keywords:** Prostate cancer, Early detection, Overdiagnosis, PSA, Screening

## Abstract

**Background:**

Prostate cancer screening depends on a careful balance of benefits, in terms of reduced prostate cancer mortality, and harms, in terms of overdiagnosis and overtreatment. We aimed to estimate the effect on overdiagnosis of restricting prostate specific antigen (PSA) testing by age and baseline PSA.

**Methods:**

Estimates of the effects of age on overdiagnosis were based on population based incidence data from the US Surveillance, Epidemiology and End Results database. To investigate the relationship between PSA and overdiagnosis, we used two separate cohorts subject to PSA testing in clinical trials (n = 1,577 and n = 1,197) and a population-based cohort of Swedish men not subject to PSA-screening followed for 25 years (n = 1,162).

**Results:**

If PSA testing had been restricted to younger men, the number of excess cases associated with the introduction of PSA in the US would have been reduced by 85%, 68% and 42% for age cut-offs of 60, 65 and 70, respectively. The risk that a man with screen-detected cancer at age 60 would not subsequently lead to prostate cancer morbidity or mortality decreased exponentially as PSA approached conventional biopsy thresholds. For PSAs below 1 ng/ml, the risk of a positive biopsy is 65 (95% CI 18.2, 72.9) times greater than subsequent prostate cancer mortality.

**Conclusions:**

Prostate cancer overdiagnosis has a strong relationship to age and PSA level. Restricting screening in men over 60 to those with PSA above median (>1 ng/ml) and screening men over 70 only in selected circumstances would importantly reduce overdiagnosis and change the ratio of benefits to harms of PSA-screening.

## Background

Overdiagnosis is a critical problem in prostate cancer screening. Men with screen-detected cancers are commonly subject to radical prostatectomy or radiotherapy, leading to persistent urinary, sexual and bowel morbidities. Such treatment is no benefit for cancers that would never have become apparent in the absence of screening. Overdiagnosis was one of the reasons why the United States Preventive Services Task Force recommended against prostate specific antigen (PSA) screening [1].

Risk of overdiagnosis varies in predictable ways. For example, a cancer diagnosis prompted by a PSA of 10 ng/ml in a healthy man in his early fifties would otherwise very likely be clinically diagnosed in his lifetime; conversely, a man in his 80s with a PSA only slightly above biopsy thresholds would most probably die of another cause before signs or symptoms led to diagnosis. Yet, there are remarkably few data on the impact of age and PSA level on overdiagnosis. Papers often report specific estimates of overdiagnosis for PSA screening as a whole [2,3]. These estimates are averages that may obscure dramatic variations in overdiagnosis risk.

We hypothesized that if we could identify factors that increase the risk of overdiagnosis, appropriate changes in screening practices could importantly shift the ratio of benefits to harms. In this paper, we examine the influence of age and PSA on prostate cancer overdiagnosis. Specifically, we sought to estimate the proportion of excess cases diagnosed in the US in the years after the introduction of the PSA-test that would have been avoided had PSA screening been restricted to younger men. We then sought to estimate the risk of biopsy detectable cancer relative to the long-term risk of prostate cancer morbidity at a given PSA for 60-year-old men, approximately the midpoint of the age range in many screening recommendations. Both excess incidence and ratio of the risk of cancer morbidity compared to biopsy detectable cancer are correlates of overdiagnosis, rather than direct estimates, and are useful primarily for comparison between groups. We hypothesized that there would be large differences in the number of excess cases by age group and risk of cancer-related morbidity by baseline PSA level.

## Methods

To investigate the association between age and excess incidence we used data from the Surveillance, Epidemiology and End Results (SEER) database. SEER 9 data derive from several geographic regions in the US, representing close to 10% of the US population. The PSA test started to be widely used as a screening test in the late 1980s, and the incidence sharply increased after that point [4]. We compared the observed incidence of prostate cancer between 1987 and 1995 with the predicted incidence without PSA screening during that time. The cutoff year of 1995 was chosen as it is often considered to constitute when incidence began to stabilize [5] and when stage shift was no longer observed in clinical practice [6]. As a sensitivity analysis, we explored other end dates.

To predict what the incidence of prostate cancer would have been without the introduction of PSA screening, we assumed that changes in incidence between 1973 and 1986 would continue through 1995. For each year in age, we created a linear regression model to predict the incidence by year of diagnosis for 1987 to 1995 on the basis of incidence 1973 to 1986. To estimate the number of excess cases for a given year and age, we simply subtracted predicted from observed cancer diagnoses and standardized to the entire US population. We then smoothed estimates by age using locally-weighted scatterplot smoothing (lowess), which can be thought of in terms of a moving average of the number of excess cases for each year of age at diagnosis. We repeated our analyses assuming that incidence after 1987 would have been similar to that for 1986 had PSA testing not been introduced. This counterfactual is supported by at least one statistical analysis of incidence trends [5].

Calculation of excess cases provides only an indirect estimate of overdiagnosis. This is for several reasons. First, some excess cases within an age stratum would not constitute overdiagnosis as a cancer might be clinically diagnosed subsequently. For instance, a man diagnosed by screening in his late 50s might present with prostate cancer symptoms in his early 70s and, therefore, would not be an overdiagnosed case. However, this effect constitutes a bias against our hypothesis that a large proportion of overdiagnosed cases occur in older men: more of the excess cases in young men would eventually be clinically diagnosed than those in older men simply because younger men live longer, providing more time for the cancer to become apparent. Second, the distribution of excess cases is partly a reflection of screening rates within age groups. If there were more excess cases in, say, men aged 70 to 75 compared to those aged 65 to 70, this may just reflect that more men in the older cohort are getting PSA tests. However, that has no bearing on the practical effect of restricting screening in older men. If most of the excess incidence is in older men, then screening fewer older men will have a disproportionate effect on overdiagnosis, irrespective of the exact mechanism underpinning the distribution of excess incidence. Finally, a precise estimate of the exact proportion of overdiagnoses in each age stratum is not the aim of the paper. We aimed only to determine if a substantial proportion of overdiagnoses were in older men; whether that proportion is 30%, 50% or 80%, our conclusion would be the same, namely that restricting screening among older men would have a large impact on overdiagnosis.

To investigate the relationship between overdiagnosis and PSA level, we used data from an unscreened representative population of men in Malmö, Sweden, who participated in the Malmö Preventive Program (MPP). The study has been previously described [7,8]. In brief, 1,162 60-year-old men, constituting 71% of the eligible population, gave blood in 1981 to 1982 and were followed to age 85. Cancer diagnoses were obtained from the Swedish cancer registry, with ascertainment of metastasis and death predominately on the basis of case notes. Using a case–control design, archived ethylenediaminetetraacetic acid (EDTA) anti-coagulated blood plasma samples from participants were thawed and analyzed using a method that has been demonstrated to give results close to what a contemporaneous PSA test would have measured [9]. The risk of clinical diagnosis (rates of PSA screening were very low during the period of the study), metastasis and mortality by age 85 for a given PSA level were calculated as described previously [10]. The Malmo cohort was close to 100% Caucasian and family history data were not available, hence neither race nor family history was entered into the analysis.

We also obtained data from two randomized trial cohorts that involved PSA testing, where patients were subject to prostate biopsy mandated by study protocols: the control arm of the Prostate Cancer Prevention Trial (PCPT) [11] and the screening arm of the European Randomized Study of Screening for Prostate Cancer (ERSPC) in Rotterdam [2]. All men in the ERSPC cohort were referred to biopsy due to an elevated PSA at their first PSA test (≥3 ng/ml) in 1993 to 1999; the PCPT cohort included men undergoing regular screening who had at least one prior low PSA (<3 ng/ml) and who were either biopsied 'for cause’ (PSA elevated above 4 ng/ml or suspicious digital rectal exam) or biopsied without indication at the end of the trial (1995 to 2003) as part of the study protocol. Both cohorts were restricted to men close to 60 years old (defined as 55 to 65), with 1,577 participants from PCPT and 1,197 from ERSPC. We used lowess methods to estimate the risk of biopsy-detectable cancer for a given PSA level. All PSA levels were standardized to the WHO calibration.

We divided the risk of cancer on biopsy from the PCPT or ERSPC cohorts by the long-term risk of clinical prostate cancer, metastasis and cancer-specific mortality in MPP. All analyses were conducted using Stata 12.0 (Stata Corp., College Station, TX, USA). This study involved reanalysis of fully deidentified data that had been taken for other purposes and previously analyzed as part of other research studies. Permission to use data from PCPT and ERSPC was obtained from the appropriate oversight entities. Data were analyzed under waivers from the Memorial Sloan-Kettering Cancer Center institutional review board.

## Results

**Figure 1:**
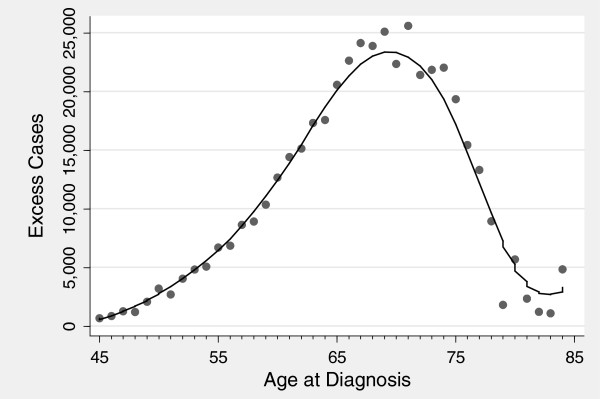
**Number of excess prostate cancer cases by age at diagnosis 1987 to 1995.** The 95% confidence interval is extremely narrow and is not shown here.

Table 1 shows the number of excess cases separately by age at diagnosis, with the results shown graphically in Figure 1. It is clear that there is a strong association between age and excess cases with low frequency in younger men (fewer than 20,000 excess cases in ages 50 to 54) compared to older men (approaching 115,000 excess cases for the 70 to 74 age group). As pointed out above, an excess case does not necessarily imply overdiagnosis, so the key analysis is the relative number of cases between groups rather than absolute numbers within each group. Table 1 shows the cumulative proportion of excess cases. This can be thought of in terms of the reduction in excess cases had screening been restricted to younger men. If testing had not been available for men 60+, 65+, or 70+ years old, excess cases would have been reduced by 85%, 68%, and 42%, respectively.

**Table 1:** Estimated number of excess cases of prostate cancer diagnosed between 1987 and 1995

**Age category**	**Observed**	**Predicted**	**Excess cases**	**95% confidence interval**	**Cumulative proportion**
45 to 49 years	10,232	4,277	5,955	5,803 to 6,106	1.3%
50 to 54 years	37,389	17,633	19,756	19,480 to 20,031	5.7%
55 to 59 years	88,783	47,407	41,376	40,977 to 41,774	15.0%
60 to 64 years	188,018	111,004	77,014	76,471 to 77,558	32.1%
65 to 69 years	311,865	195,602	116,263	115,596 to 116,930	58.1%
70 to 74 years	351,991	238,803	113,188	112,530 to 113,846	83.3%
75 to 79 years	277,274	218,476	58,798	58,324 to 59,273	96.5%
80 to 85+ years	261,895	246,002	15,893	15,646 to 16,140	100%

Our findings were not importantly changed in sensitivity analysis. There was little effect of assuming a stable incidence in the absence of screening (for example, 47% of cases diagnosed in men older than 70 years). When the time period is extended out to the year 2000, the relationship between age and excess cases is moderated, with 78%, 58% and 35% of cases in men older than 60, 65 and 70 years. A possible explanation for this effect is that men overdiagnosed in their 70s during the early part of the 1990s are not at risk for overdiagnosis in their 80s towards the end of the decade. As a final sensitivity analysis, we changed the incidence at age 50 to 59 years old between 1987 to 1995 so that it matched that reported in 2005 to 2009, keeping the incidence at other ages unchanged. Even under this very conservative assumption, the proportion of excess cases in men 60, 65, and 70 years old was 68%, 54%, and 33%, respectively.

**Figure 2:**
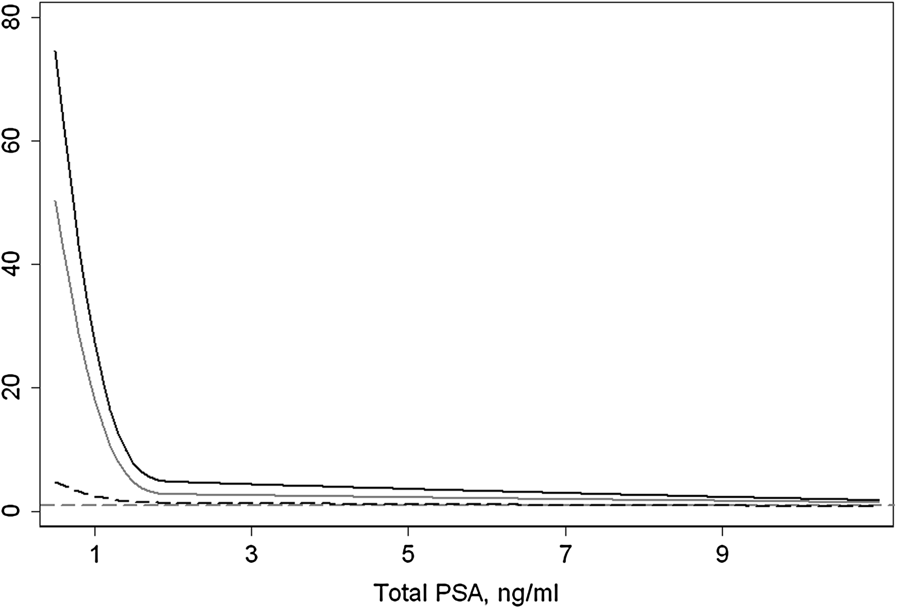
**Risk of biopsy detectable cancer in a screened population divided by the 25 year risk of death from prostate cancer (solid black line), distant metastasis (solid grey line), and clinical diagnosis of prostate cancer (dashed black line) in an unscreened cohort, by PSA level at age 60.** The dashed grey line at a ratio of 1 is included as reference. Risk of biopsy detectable cancer was obtained from the PCPT. The clinical endpoints were obtained from the Malmö cohort. PCPT, Prostate Cancer Prevention Trial; PSA, prostate specific antigen.

Table 2 gives absolute risk by PSA level at age 60 for biopsy detectable cancer in the screened cohorts versus that of clinical cancer events within 25 years in the unscreened cohort. The long-term risk of metastasis and cancer-specific death remains low at PSA levels below age median (≤1 ng/ml) and increases much more rapidly for PSAs >1 ng/ml compared to the risk of screen- or clinically-detected cancer. For example, risk of death from disease rises more than 10-fold between PSAs of 1 and 4 ng/ml, compared to only about a 1.5-fold rise for the risk of a positive biopsy. This relationship is shown in Table 3 and Figure 2. Patients with PSA levels below common prostate biopsy thresholds at age 60 have a much greater probability of a positive biopsy than of dying from prostate cancer by age 85. For example, at a PSA of 1 ng/ml, a 60-year-old man is 29 times more likely to have a positive biopsy than to die from cancer. This ratio rises rapidly to greater than 100 as PSA falls below 1, but is markedly reduced for PSAs above 3 ng/ml. Results for higher PSAs are fairly comparable between men subject to repeat screening (PCPT) and those undergoing an initial PSA test (ERSPC). The ratio between risk of biopsy-detectable cancer and risk of metastasis falls slightly more rapidly in the placebo-treated controls of the PCPT cohort, likely because, in intensively screened individuals, risk of a positive biopsy does not importantly rise as PSA increases above biopsy thresholds [12].

**Table 2:** Absolute risk of biopsy detected cancer compared to the 25 year-risk of clinical prostate cancer endpoints

**PSA (ng/ml)**	**Clinical diagnosis**	**Distant metastasis**	**Cancer-specific mortality**	**Biopsy detected cancer (PCPT)**	**Biopsy detected cancer (ERSPC, Rotterdam)**
<1.0	4.5 (2.9, 6.1)	0.6 (<0.1, 1.2)	0.2 (<0.1, 0.6)	12.2 (10.1, 14.6)	-
0.5	3.8 (2.2, 5.4)	0.3 (0.1, 0.6)	0.1 (<0.1, 0.2)	10.1 (8.2, 11.7)	-
1.0	6.1 (5.0, 7.3)	1.2 (0.6, 1.7)	0.6 (0.2, 1.1)	16.9 (15.8, 18.2)	-
2.0	13.1 (11.2, 14.2)	4.6 (3.6, 5.1)	3.8 (2.9, 4.2)	21.5 (20.2, 23.0)	-
3.0	16.8 (15.3, 20.3)	6.6 (5.6, 8.4)	5.5 (4.8, 7.3)	22.2 (20.5, 23.9)	15.4 (8.1, 23.6)
4.0	19.6 (17.8, 26.9)	8.6 (7.3, 12.4)	7.2 (6.1, 11.3)	23.2 (20.3, 25.4)	18.6 (16.3, 20.8)
5.0	21.9 (19.6, 32.9)	10.3 (8.7, 16.4)	8.6 (7.1, 15.2)	23.5 (19.5, 26.5)	22.1 (20.3, 23.6)
7.5	28.2 (24.0, 49.7)	14.9 (12.2, 27.2)	12.4 (10.0, 25.9)	24.6 (16.8, 30.2)	29.8 (27.4, 31.6)
10.0	33.6 (27.9, 65.0)	19.1 (15.4, 37.4)	15.9 (12.4, 36.0)	25.6 (13.1, 33.9)	36.1 (32.9, 39.1)

The data compare an unscreened Swedish cohort (MPP) with two screened cohorts (PCPT and ERSPC) by PSA level at age 60.

Data are given as percent risk with 95% confidence interval. Data are not given for PSA <3 ng ml in the ERSPC cohort as biopsy was restricted to men with elevated PSA. ERSPC**,** European Randomized Study of Screening for Prostate Cancer; MPP, Malmo Preventive Program; PCPT, Prostate Cancer Prevention Trial; PSA, prostate specific antigen.

**Table 3:** Relative risk of biopsy detected cancer compared to the 25 year-risk of clinical prostate cancer endpoints

**PSA (ng/ml)**	**PCPT (repeat screening)**			**ERSPC, Rotterdam (initial PSA test)**		
	**Clinical diagnosis**	**Distant metastasis**	**Cancer-specific mortality**	**Clinical diagnosis**	**Distant metastasis**	**Cancer-specific mortality**
<1.0	2.7 (1.9, 4.2)	21.6 (9.6, 69.5)	64.9 (18.2, 72.9)	-	-	-
0.5	2.7 (1.8, 4.5)	38.0 (15.2, 192.3)	153.4 (48.2, 219.7)	-	-	-
1.0	2.8 (2.3, 3.5)	14.5 (9.7, 27.2)	28.8 (15.4, 92.1)	-	-	-
2.0	1.6 (1.5, 2.0)	4.7 (4.2, 6.1)	5.7 (5.1, 7.5)	-	-	-
3.0	1.3 (1.1, 1.5)	3.4 (2.6, 4.0)	4.0 (3.0, 4.7)	0.9 (0.5, 1.4)	2.3 (1.2, 3.6)	2.8 (1.4, 4.2)
4.0	1.2 (0.8, 1.3)	2.7 (1.8, 3.2)	3.2 (2.0, 3.9)	0.9 (0.7, 1.1)	2.2 (1.5, 2.6)	2.6 (1.6, 3.1)
5.0	1.1 (0.7, 1.2)	2.3 (1.3, 2.8)	2.7 (1.5, 3.4)	1.0 (0.7, 1.1)	2.2 (1.3, 2.6)	2.6 (1.4, 3.1)
7.5	0.9 (0.4, 1.1)	1.7 (0.8, 2.1)	2.0 (0.8, 2.7)	1.1 (0.6, 1.3)	2.0 (1.1, 2.4)	2.4 (1.1, 3.0)
10.0	0.8 (0.3, 1.0)	1.3 (0.5, 1.8)	1.6 (0.5, 2.3)	1.1 (0.5, 1.3)	1.9 (1.0, 2.4)	2.3 (1.0, 2.9)

The data compare an unscreened Swedish cohort (MPP) with two screened cohorts (PCPT and ERSPC) by PSA level at age 60.

Data are given as percent risk with 95% confidence interval for two screened cohorts separately. Data are not given for PSA <3 ng/ml in the ERSPC cohort as biopsy was restricted to men with elevated PSA. ERSPC**,** European Randomized Study of Screening for Prostate Cancer; PCPT, Prostate Cancer Prevention Trial; PSA, prostate specific antigen.

Across PSA levels, the risk of biopsy-detectable cancer is similar to the risk of a clinical diagnosis of cancer within 25 years. The ratio of these two risks ranges from slightly above 2 for low PSAs to close to 1 for PSAs above common biopsy thresholds, such as 4 ng/ml. We assume that most men with elevated PSA at age 60 and who subsequently develop prostate cancer would have had a positive biopsy had they been biopsied at 60. If such an assumption is correct, these results suggest that a high proportion of 60 year olds with screen-detected cancer following an elevated PSA will develop prostate cancer that is detectable by symptoms over the course of the subsequent 25 years, that is, the rate of overdiagnosis is low.

## Discussion

We analyzed population-based data on prostate cancer incidence and found that an important majority of excess cases diagnosed in the first few years after the introduction of PSA testing occurred in men older than 60. We then estimated the risk at age 60 for prostate biopsy-detectable cancer in two different PSA-screened population-based cohorts and compared this risk to that of long-term metastasis and death from prostate cancer in an unscreened population. The risk that a biopsy detectable cancer would not lead to cancer-related morbidity or mortality increased exponentially as PSA fell below conventional biopsy thresholds.

There are two major clinical implications of our findings. First, more selective screening of men older than 60 is justified. We found that a clear majority of excess cases are diagnosed in men older than 60, yet there is randomized evidence from the European trial [2] that screening reduces mortality for men in their 60s. As such, it would appear unwise to recommend that screening be terminated at age 60 for all men. An alternative would be to restrict screening to men with PSA levels above 1 ng/ml (WHO calibration), close to the median. Men with PSA <1 ng/ml – approximately 50% of the population – can be told that if they continue to be screened, any prostate cancer thereby detected is unlikely to harm them and that if they elect to be treated they will likely be subjecting themselves to overtreatment. The ERSPC found evidence that screening is not of benefit for men who start at age 70 or older, with the lower bound of the 95% CI excluding the central estimate for risk reduction for men younger than 70 [2]; a subsequent modeling study reported that any decreases in mortality associated with screening men older than 70 were offset by overdiagnosis [13]. Restricting screening in men in their 70s to a small group with excellent health and above average PSA would likely reduce overdiagnosis considerably without any substantive effect on mortality.

The second implication of our findings is that it becomes hard to justify prostate biopsy in men with PSA below typical thresholds for biopsy, such as 3 or 4 ng/ml. It has been estimated that about one in seven diagnoses occur in men with PSA below 4 ng/ml [14], constituting about 35,000 cases a year. Although use of a lower PSA threshold may be justifiable in younger men, such as those below 50 years old, in clinical practice, older men with low PSA are often subject to biopsy because of a positive digital rectal exam, rapid increase in PSA, low ratio of free-to-total PSA or family history [11,12]. Such indications would only be justified if they dramatically raised the risk of aggressive cancer. There is no clear evidence that this is the case.

Several lines of evidence from the literature support our overall findings. First, the strong association between overdiagnosis and age is supported by consideration of life expectancy data. In the studies with appropriately long follow-up, lead time has been estimated to average around 12 years [15,16]. For instance, in the Malmö cohort used for this paper, the mean time to clinical diagnosis was 11.8 years among men who were subsequently diagnosed with cancer and who had a baseline PSA ≥3 ng/ml at age 60 [15]. From the Social Security Life Tables it can be calculated that the probability of death within 12 years is 21% for a 60 year old but 45% for a 70 year old. This means that, for a group of 200 men with screen-detected cancer, half 60 years old and half 70 years old, 66 men would die before they would be expected to be clinically diagnosed. Of these, 45, close to 70%, would be in the older age group. These data can also be used to support our finding that, in 60-year-old men with PSAs above biopsy thresholds, most screen-detected cancers would eventually lead to a clinical diagnosis, as close to 80% of men survive longer than the mean lead time. Moreover, the Malmö cohort does not stand alone in finding that PSA is strongly predictive of prostate cancer mortality in unscreened populations. Numerous other studies have shown associations between baseline PSA and long-term prostate cancer outcomes [17-22].

PSA screening can only reduce mortality in that it leads to curative treatment. Two trials have compared surgery with conservative management for prostate cancer [23,24] and both report a decreased effect of treatment in older men. In one analysis [25], differences between surgery and conservative treatment started to decrease around age 65 with little benefit for men older than 70. These studies indicate that the effects of treatment diminish with age, suggesting that men older than 70 should only be considered for screening if they are at higher than average risk for prostate cancer mortality and lower than average risk for other cause mortality. Evidence that a risk stratification approach, based on PSA, would improve screening outcomes is provided by a reanalysis of the ERSPC data. Van Leeuwen *et al*. evaluated the effects of PSA-based screening on men in reference to the PSA-level measured at their first screen. Their results demonstrate that had men with a PSA <2 ng/ml at baseline been excluded from further screening, the number of men needing to be screened and diagnosed to prevent one death would be reduced by 90% and 50%, respectively. Results for a PSA cut-off of 1 ng/ml are not reported. These findings are of particular relevance to our recommendations, as the median age in the screened group was 61 [26].

One study using SEER data came to a quite different conclusion from the current paper. Welch and Albertsen reported that excess incidence 'which must represent overdiagnosis’ was 'particularly dramatic for younger men’ [27]. There are two major problems with this conclusion. First, it focuses on relative rather than absolute increase in diagnoses. So, for example, the reported seven-fold increase in men younger than 50 constitutes only 8 additional cases per 100,000. A relative seven-fold increase sounds large, suggesting that public health efforts might focus on reducing screening in this age group, whereas the absolute increase demonstrates that such efforts would not have an important impact at the population level. Second, the authors look at an extended period of PSA screening, leading to age-related artifacts. For instance, they state that in men older than 80, 'incidence declined dramatically between 1986 and 2005’. This is because some men who would have been clinically diagnosed in their 80s in the years 2000 to 2005 were screen-detected at an earlier age. It would be entirely unsound to use this finding to suggest that use of PSA in older men does not lead to overdiagnosis.

It is worth considering differences both between the different cohorts within the study and between these study cohorts and contemporary patients. First, the biopsy cohorts and the SEER population sample predominately involved 6-core biopsy rather than the more extended biopsy schemes typical in current practice. This suggests that we may have underestimated the risk of screen-detected cancer. Second, prostate cancer mortality has historically been higher in Sweden – around 5% of male deaths compared to less than 3% in the US [28]. Moreover, recent advances in treatment have led to improvements in survival leading to a lower risk of death [29]. This suggests that we may have overestimated the risk of death. The effect of any such misestimation – underestimating cancer incidence, overestimating mortality risk - would be to exacerbate the difference in risk by PSA levels: men with a low PSA would be even more likely to have cancer detected and even less likely to die without screening.

A clear limitation of our paper is that we are measuring excess incidence, which is only a correlate of overdiagnosis. Yet, overdiagnosis by its very nature is a counterfactual – that a patient would die without a diagnosis if not for screen detection – and, therefore, cannot be directly observed. Moreover, an increase in incidence, even if only temporary, is a prerequisite of screening: a screening test associated with zero overdiagnosis would still raise incidence after clinical implementation, even though incidence rates would subsequently fall below prior levels. That said, a persisting increase in incidence, as seen in the US, is clear evidence of overdiagnosis; comparably, the gross disparities we have seen in the relative number of excess cases by age clearly suggests that restricting PSA screening in older men would importantly shift overdiagnosis rates.

## Conclusions

In conclusion, we provide evidence that correlates of overdiagnosis are strongly related to age and PSA level. Overdiagnosis appears to be predominately a problem of older men and those with lower PSAs. The ratio of benefits to harms for prostate cancer screening could be improved by restricting screening in men older than 60 to those with PSA >1 ng/ml and restricting screening in men older than 70 to those in excellent health and higher PSA levels.

## Competing interests

Hans Lilja holds patents for free PSA, hK2 and intact PSA assays and with Andrew Vickers, is named on an application for a statistical method to predict the result of prostate biopsy. The other authors declare that they have no competing interests.

## Authors’ contributions

The concept for the study was developed by AJV and HL. Statistical analysis was conducted by DDS, CAS and EV. Advice on the analysis and interpretation was provided by DU, MJR, IT, EAMH, HDK and PTS. AV wrote the first draft of the manuscript. All authors read and approved the final manuscript.
